# Supplementary material for: The Construction and Exploration of a Comprehensive MicroRNA Centered Regulatory Network in Foxtail Millet (Setaria italica L.)
Source: Front Plant Sci. 2022 May 6;13:848474. doi: 10.3389/fpls.2022.848474 (PMC9121102; doi:10.3389/fpls.2022.848474)

T=Seita.7G072700.1_Q=Sit−miR396a_S=500

category=2_p=0.999311039062082

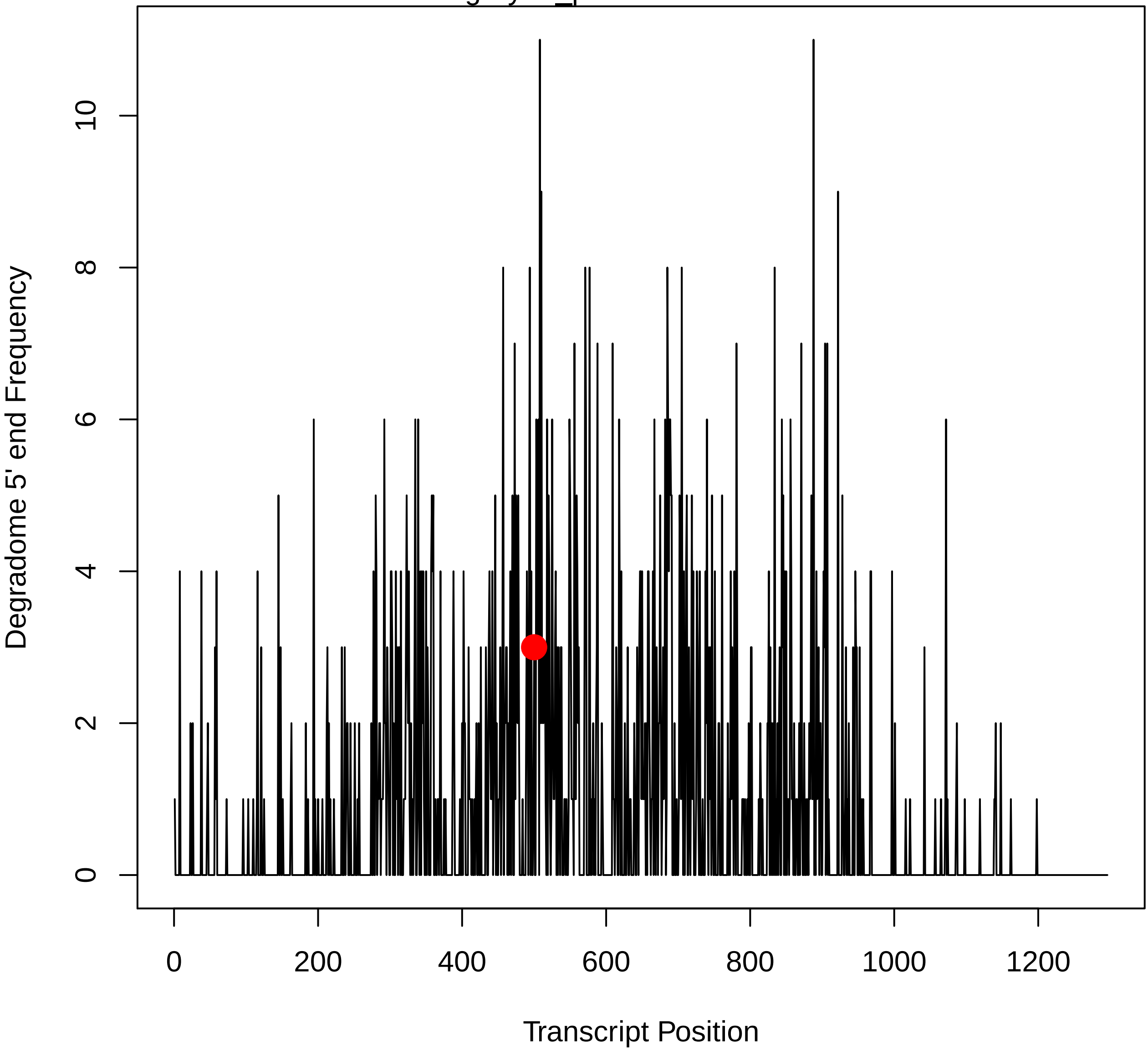

Supplement: Supplementary file 4 [file Data_Sheet_4.zip › Sit-miR396a_Seita.7G072700.1_500_TPlot.pdf]
